# Supplementary material for: Polarization curling and flux closures in multiferroic tunnel junctions
Source: Nat Commun. 2016 Nov 16;7:13484. doi: 10.1038/ncomms13484 (PMC5116095; doi:10.1038/ncomms13484)
Supplement: Supplementary Information — Supplementary Figures 1-6 [file ncomms13484-s1.pdf]

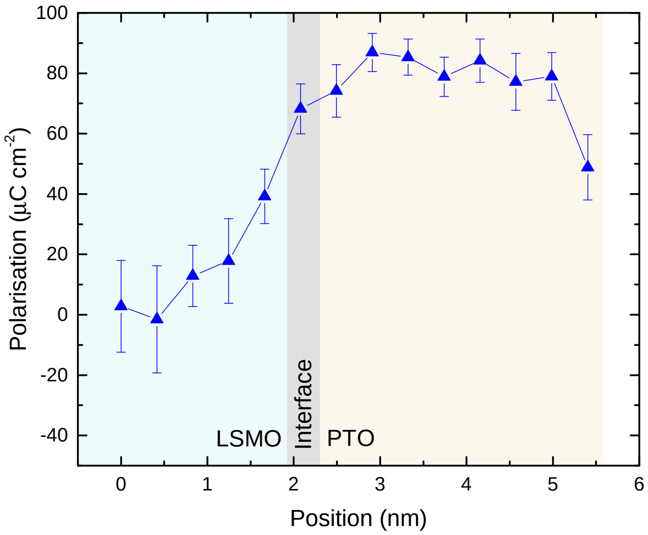

Supplementary Figure 1 | Tunnel junction polarisation profile. Profile of average polarisation across the thickness of the 9 unit cells film, showing the interface between the PTO and LSMO. Data was acquired by averaging rows of unit cells, error bars are the corresponding standard errors of the means.

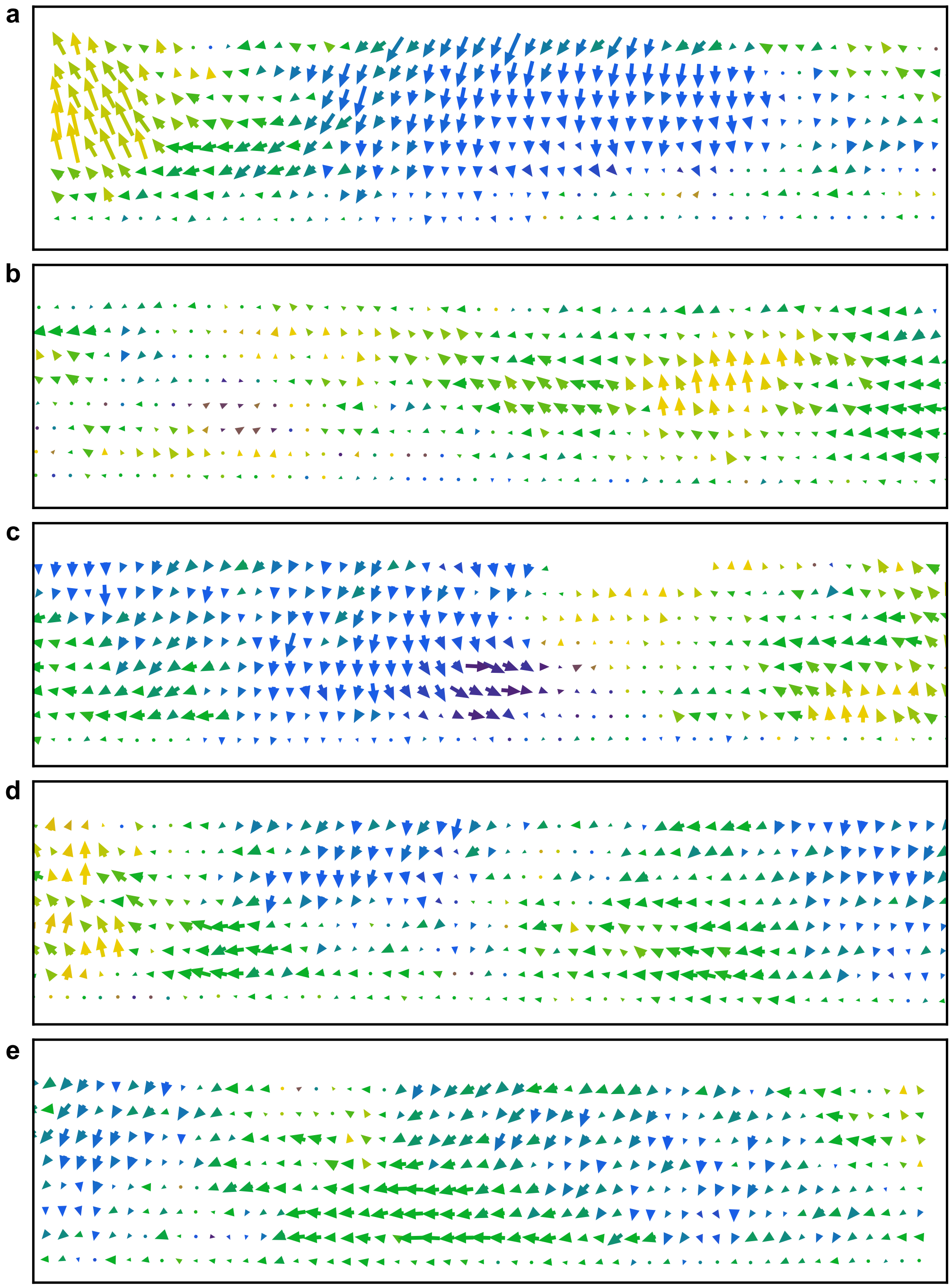

Supplementary Figure 2 | Full polarisation map of 6 unit cells thick tunnel junction. Quiver plots of Dipoles from the 6 unit cells PTO layer spanning a ∼75 nm range. The right hand side of each image is continued from the left hand side of the following image.

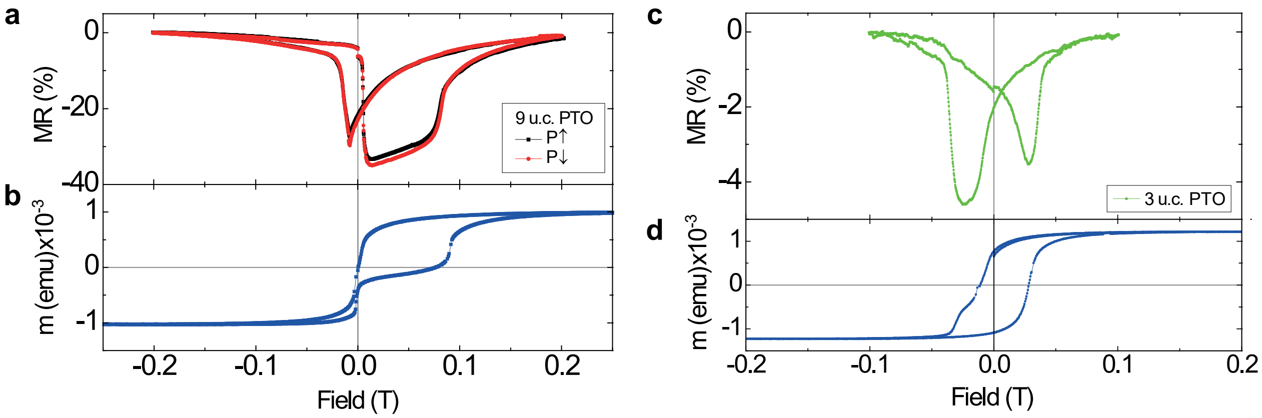

Supplementary Figure 3 | Magnetic measurements. (**a**) TMR and (**b**) total magnetic moment for the 9 unit cells (u.c.) thick PTO layers. (**c**) TMR and (**d**), total magnetic moment for the 3 u.c. thick PTO layers. TMR was measured from 40×40 µm^2 area devices and total magnetic moment from 5×5 mm^2 area samples. **a** shows the TMR with both polarisation pointing towards the LSMO (**P**↓) and Co (**P**↑) electrodes. All measurement made at T=10 K.

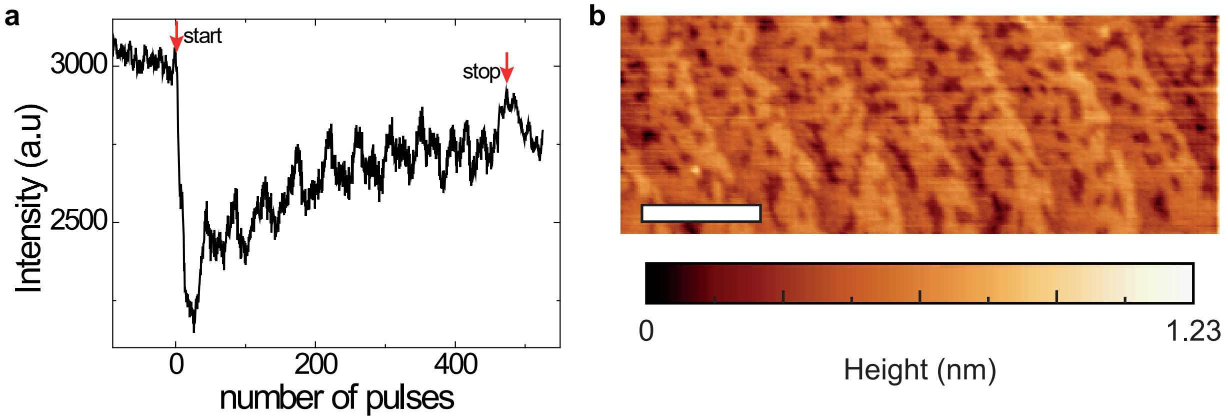

Supplementary Figure 4 | Growth control and topography. (**a**) In-situ RHEED oscillations from the growth of the 9 unit cells (u.c.) sample. (**b**) AFM topography from a 0.7×2 μm^2 area of the 9 u.c. thick PTO layer before Co deposition. Scale bar, 400 nm.

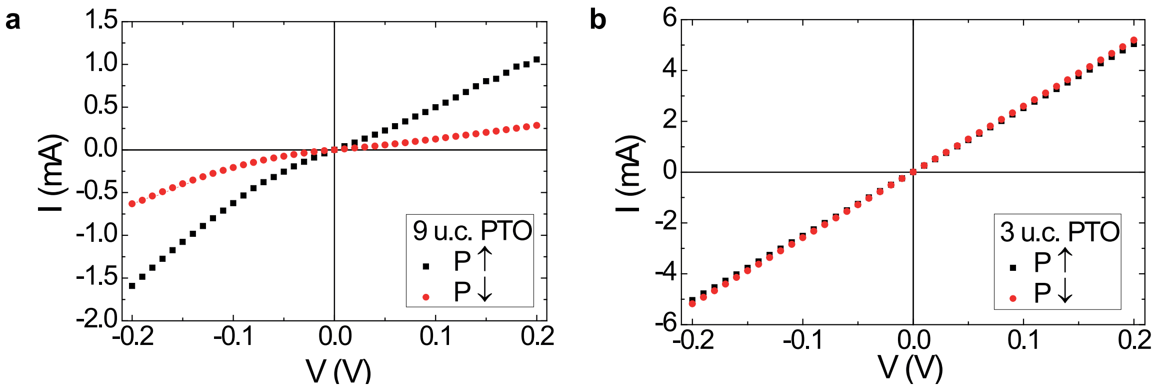

Supplementary Figure 5 | I-V characteristics. Current-voltage (I-V) characteristics measured at T=10 K for a 9 unit cells (u.c.) (**a**) and 3 u.c. (**b**) thick PTO barrier. Values are measured after switching polarisation towards LSMO (**P**↓) and towards Co (**P**↑).

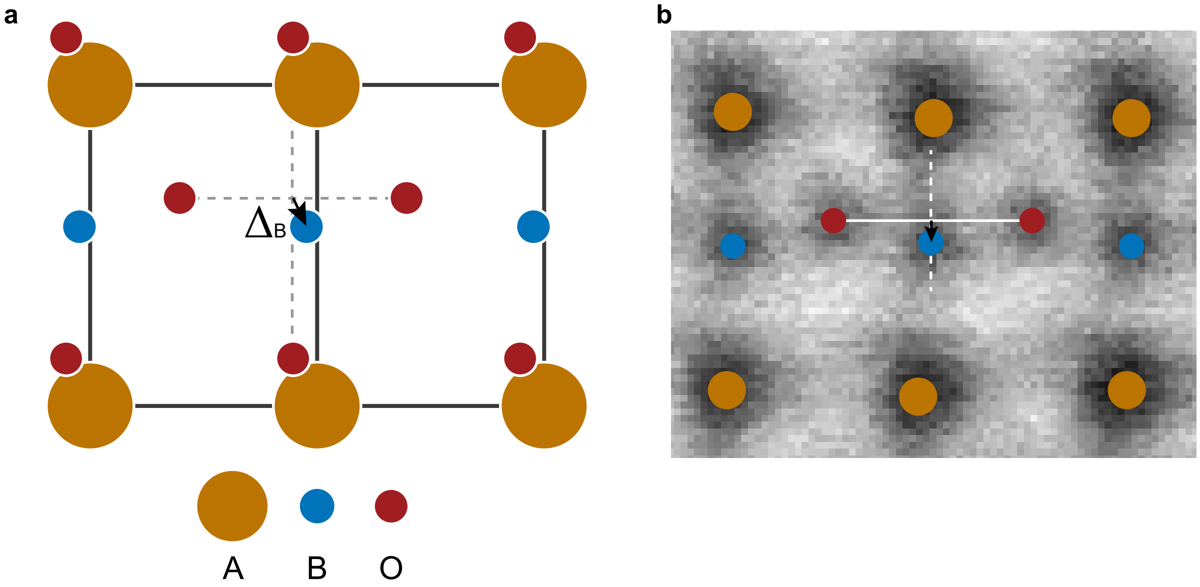

Supplementary Figure 6 | Displacement measurements. (**a**) Schematic of the measured dipole displacements, Δ_B, (black arrow) in an ABO_3 perovskite, viewed along the [110] direction. Dashed lines define the centre of the oxygen octahedra. (**a**) Dipole as measured from an experimental image of PTO. The solid white line joins the two nearest oxygen positions with the dashed line bisecting it to give the octahedra centre.
